# A multiplex pneumonia panel for diagnosis of hospital-acquired and ventilator-associated pneumonia in the era of emerging antimicrobial resistance

**DOI:** 10.3389/fcimb.2022.977320

**Published:** 2022-10-12

**Authors:** Anupop Jitmuang, Soravit Puttinad, Sivaporn Hemvimol, Siri Pansasiri, Navin Horthongkham

**Affiliations:** ^1^ Department of Medicine, Faculty of Medicine Siriraj Hospital, Mahidol University, Bangkok, Thailand; ^2^ Department of Medicine, Saraburi Hospital, Saraburi, Thailand; ^3^ Saraburi Hospital Research Center, Saraburi Hospital, Saraburi, Thailand; ^4^ Department of Microbiology, Faculty of Medicine Siriraj Hospital, Mahidol University, Bangkok, Thailand

**Keywords:** antimicrobial resistance, hospital acquired pneumonia, ventilator - associated pneumonia, multiplex real time PCR, commercial multiplex PCR

## Abstract

**Background:**

Antimicrobial resistance (AMR), including multidrug (MDR) and extensively drug-resistant (XDR) bacteria, is an essential consideration in the prevention and management of hospital-acquired pneumonia (HAP) and ventilator-associated pneumonia (VAP). In the AMR era, the clinical utility of the BioFire FilmArray Pneumonia Panel Plus (BFPP) to diagnose HAP/VAP has not been thoroughly evaluated.

**Methods:**

We enrolled adult hospitalized patients with HAP or VAP at Siriraj Hospital and Saraburi Hospital from July 2019–October 2021. Respiratory samples were collected for standard microbiological assays, antimicrobial susceptibility testing (AST), and the BFPP analysis.

**Results:**

Of 40 subjects, 21 were men. The median duration of HAP/VAP diagnoses was 10.5 (5, 21.5) days, and 36 endotracheal aspirate and 4 sputum samples were collected. Standard cultures isolated 54 organisms—*A. baumannii* (37.0%), *P. aeruginosa* (29.6%), and *S. maltophilia* (16.7%). 68.6% of Gram Negatives showed an MDR or XDR profile. BFPP detected 77 bacterial targets—*A. baumannii* 32.5%, *P. aeruginosa* 26.3%, and *K. pneumoniae* 17.5%. Of 28 detected AMR gene targets, CTX-M (42.5%), OXA-48-like (25%), and NDM (14.3%) were the most common. Compared with standard testing, the BFPP had an overall sensitivity of 98% (88-100%), specificity of 81% (74-87%), positive predictive value of 60% (47-71%), negative predictive value of 99% (96-100%), and kappa (*κ*) coefficient of 0.64 (0.53-0.75). The concordance between phenotypic AST and detected AMR genes in *Enterobacterales* was 0.57. There was no concordance among *A. baumannii*, *P. aeruginosa*, and *S. aureus*

**Conclusions:**

The BFPP has excellent diagnostic sensitivity to detect HAP/VAP etiology. The absence of *S. maltophilia* and discordance of AMR gene results limit the test performance.

## Introduction

Hospital-acquired pneumonia (HAP) and ventilator-associated pneumonia (VAP) are leading causes of hospital-acquired infection (HAI). Several bacteria that cause HAI are evolving resistance to commonly used antibacterial agents (Yungyuen et al., 2021). The rate of antimicrobial resistance (AMR) in HAI is increasing in Thailand (Yungyuen et al., 2021), and infection with AMR bacteria contributes to poor health and economic outcomes in hospitalized patients (Thamlikitkul et al., 2020). Among bacteria causing HAP and VAP, multidrug-resistant (MDR) and extensively drug-resistant (XDR) bacteria have become common in our institute. Our recent study demonstrated carbapenem-resistant and XDR *Acinetobacter baumannii* (25-50%), MDR *Pseudomonas aeruginosa* (30-35%), extended-spectrum cephalosporin-resistant *Klebsiella pneumoniae* (20-25%), *Stenotrophomonas maltophilia* (8-20%), and methicillin-resistant *Staphylococcus aureus* (5-10%) in HAP and VAP patients (Jitmuang et al., 2020). Conventional microbiological tests have limited sensitivity and specificity to diagnose HAP and VAP, and it usually takes several days to know the final antimicrobial susceptibility profile (Kalil et al., 2016). Thus, the ability to rapidly identify bacteria and the presence of antimicrobial resistance causing HAP/VAP is important in this era of antimicrobial resistance.

The BioFire FilmArray pneumonia panel plus ((bioMérieux, Marcy-l’Etoile, France) uses a multiplex real-time PCR assay to identify 15 bacterial, 3 atypical bacterial, 9 viral, and 7 AMR gene targets with an approximately 1-hour turnaround time (Serigstad et al., 2022). Previous studies have reported that the BioFire FilmArray pneumonia panel (BFPP) can increase the positivity rate by 30-60% compared with conventional microbiological diagnoses (Buchan et al., 2020; Gastli et al., 2021; Zacharioudakis et al., 2021; Serigstad et al., 2022). The sensitivity and specificity to detect bacterial targets were 90-100%, depending on the targeted organisms, sample type, and patient setting (Buchan et al., 2020; Murphy et al., 2020; Webber et al., 2020; Ginocchio et al., 2021). BFPP has also shown good sensitivities (90-98%) and specificities (76-98%) to identify the etiology of HAP/VAP (Lee et al., 2019; Yoo et al., 2020; Mitton et al., 2021). However, AMR gene detection by the BFPP cannot be connected to the corresponding bacterial target, mainly when several bacteria are detected (BioFire, 2021). Multiple pathogens are often found in a single sample from HAP/VAP patients (Jitmuang et al., 2020). Little is known about the diagnostic efficacy and limitations of the BFPP assay to diagnose HAP/VAP in a setting where MDR and XDR pathogens are prevalent, and co-infections are common. Therefore, we aimed to evaluate the diagnostic efficacy and limitations of the BFPP assay to detect the etiology of HAP/VAP and associated AMR.

## Materials and methods

We conducted a multicenter prospective observational study of adult subjects aged ≥ 18 years who developed HAP or VAP from July 2019 to October 2020 at the Department of Medicine, Faculty of Medicine Siriraj Hospital, Bangkok, or the Department of Medicine, Saraburi Hospital, Saraburi Province, Thailand. HAP or VAP was diagnosed according to the American Thoracic Society (ATS) and Infectious Diseases Society of America (IDSA) guideline [American Thoracic Society (ATS) and Infectious Diseases Society of America (IDSA) (2005)]. HAP was defined as pneumonia occurring at ≥ 48 hours after hospital admission, and VAP was defined as pneumonia occurring 48 hours or more after endotracheal intubation. The diagnosis of HAP and VAP requires abnormal radiological findings of new or progressed pulmonary infiltrate(s) plus clinical signs of infection, such as a new onset of fever (≥ 38 °C), increased sputum production and/or sputum changed to more purulent, peripheral leukocytosis, and decreased oxygenation or requiring oxygen supplement therapy. This study was approved by the Scientific Ethics Committee, Siriraj Institutional Review Board (COA *Si* 673/2020). Patient demographic data, comorbidities, medical history, reasons for hospitalization and type of in-patient ward, the onset of HAP/VAP, type of sample, microbiological results, and treatment outcomes were collected. In addition, in duplicates, expectorated sputum or endotracheal aspirate samples were collected; one sample was sent to the clinical microbiology laboratory of the responsible hospital for a conventional microbiological diagnosis, and another was sent to the Molecular Diagnostic Unit, Department of Microbiology, Siriraj Hospital for BFPP testing. Samples for BFPP testing collected from subjects in the Saraburi Hospital were stored at 2-4 °C in the hospital microbiology laboratory for 1-3 days before transfer to Siriraj Hospital.

### Conventional microbiological diagnoses

All expectorated sputum samples were evaluated for quality by direct Gram stain. Sputum samples that exhibited abundant squamous epithelial cells or squamous epithelial cells ≥ 10/low power field (10X) were not sent for bacterial culture due to potential oral microbiota contamination. Endotracheal aspirate samples, usually collected by deep tracheal suction, were accepted for bacterial culture. All samples were inoculated onto sheep blood, MacConkey, and chocolate agars and streaked with the standard 4-quadrant technique to isolate colonies for the semi-quantitative count. Agar plates were incubated at 35°C in a 5% CO_2_ atmosphere and examined daily for bacterial isolate growth. Bacterial identification and quantification of the isolates were performed after 18-24 hours of incubation. According to the local protocol, a combination of conventional biochemical testing and automated phenotypic identification systems (Vitek-2) (bioMérieux, Durham, NC) was used to identify the potential bacterial pathogen. Growth of each isolate was reported semi-quantitatively; *rare*, ≤ 10 colonies in the first quadrant; *few*, > 10 colonies in the first quadrant; *moderate*, growth of colonies from the first into the second quadrant; and *numerous*, extended growth of colonies from the first quadrant into the third or fourth quadrant. Growth of oral microbiota without isolation of a significant pathogen was reported as normal microbiota. Antimicrobial susceptibility tests (ASTs) were performed using the disk diffusion method and commercial broth microdilution methods or Vitek-2 (bioMérieux, Durham, NC) based on the type of isolate and the local laboratory protocol. The Clinical and Laboratory Standards Institute (CLSI) interpretative breakpoint criteria were used to interpret and define the phenotypic AST profile of the isolate. The phenotypic AST profiles were defined as the following; non-MDR—isolate susceptible to all antimicrobials or non-susceptible to only a few agents from several classes of antimicrobials; extended-spectrum cephalosporin-resistant (ESCR)*—Enterobacterales* isolate resistant to at least one agent in extended-spectrum cephalosporins; MDR—isolate non-susceptible to at least one agent in three or more classes of antimicrobials; carbapenem-resistant—isolate non-susceptible to at least one agent in carbapenem group; XDR—isolate susceptible to only 1-2 agents in one or two classes of antimicrobials (Magiorakos et al., 2012).

### BioFire FilmArray pneumonia panel testing

BioMérieux Thailand Ltd, for this study, supplied forty BFPP test kits, and samples were tested according to the manufacturer’s instructions (BioFire, 2021). The staff responsible for BFPP testing did not know the results of the conventional microbiological testing. An individually packaged BFPP pouch was placed into the FilmArray pouch loading station. The sample injection vial and hydration injection vial were placed into the pouch loading station, and the provided buffer solution was added to the sample injection vial. The provided sample swab was used to transfer 1-2 mL of sputum or endotracheal aspirate sample to be mixed with buffer in the sample injection vial. Then, the sample mix was loaded into the BFPP pouch, which in turn was loaded into the FilmArray instrument (the FilmArray 2.0 system), where automated nucleic acid extraction, multiplex PCR, and post-amplification analysis were performed. At the end of each testing, the BFPP result reported a bacterial target in the copy number of specific bacterial genomes per mL (copies/mL) or a bin result in the sample, such as not detected or < 10^3.5^, 10^4^, 10^5^, 10^6^, and ≥ 10^7^ copies/mL. Meanwhile, viral and AMR gene targets were reported as “detected” or “not detected” based on an individual corresponding assay result. It is noted that CTX-M, IMP, KPC, NDM, and VIM genes are reported for all associated Gram Negatives. In contrast, the report of the OXA-48-like gene is restricted to the *Enterobacterales* Gram-negative group only when detected. *mecA/C* and MREJ genes are reported when *S. aureus* is detected.

### Definitions

#### Concordance and discordance between conventional phenotypic AST and AMR gene detection

Concordance was present when the AMR gene result was consistent with the phenotypic AST profile; discordance was present when the AMR gene detection and the phenotypic AST profile were different. Conventional phenotypic AST and genotypic AMR testing may have discrepancies (Yee et al., 2021). Negative results of the AMR genes do not determine susceptibility to corresponding classes of antimicrobials since other AMR mechanisms may exist, and those mechanisms are not fully included in the molecular panel. When AMR gene results are positive, such as CTX-M and carbapenemase genes, the BFPP assay cannot be connected to the bacterial target, mainly when several bacteria are detected (BioFire, 2021).

### Statistical analysis

Detection of the bacterial target causing HAP/VAP was compared to the conventional culture to calculate sensitivity, specificity, positive predictive values (PPV), and negative predictive values (NPV) with 95% confidence intervals. Meanwhile, positive and negative percent agreement with 95% confidence intervals were calculated when comparing the bacterial target and non-reference standard, such as Gram stain finding. In addition, an agreement between the two methods was evaluated using Cohen’s kappa (*κ*) coefficient. Agreement was classified into very good (*κ* = 0.81-1), good (*κ* = 0.61-0.80), moderate (*κ* = 0.41-0.60), fair (*κ* = 0.21-0.40), and poor (*κ* = 0.00-0.20). The AMR gene target detected by the BFPP was compared to the phenotypic AST profile, whereas there might be potential discrepancies between the two tests described above. Therefore, the *κ* coefficient and concordance between these two results were analyzed in place of evaluation of the diagnostic efficacy. In case of discordant results, medical charts and relevant clinical findings, such as any previous or current antimicrobial use and microbiological findings and procedures, were reviewed by investigators to arrive at a final determination.

## Results

Forty hospitalized subjects with HAP/VAP were enrolled: 33 (82.5%) from Siriraj Hospital and 7 (17.5%) from Saraburi Hospital. Forty sets of respiratory samples, including 36 (90%) endotracheal aspirates and four (10%) expectorated sputum samples, were collected and sent for conventional microbiological testing and the BFPP assay. **Table 1** summarizes the results of conventional microbiological testing, phenotypic AST profile, the BFPP assay detection, antimicrobial treatment, and outcomes.

**Table 1:** Results of the standard conventional microbiological testing, antimicrobial susceptibility testing profile, the BioFire FilmArray pneumonia panel plus, and antimicrobial treatment and outcomes.

Sample No.	Site of sample collection	Gram stain finding	Culture result	Phenotypic AST profile of isolates	BFPP detection (Bin results, copies/mL)	AMR gene detection	Antimicrobial treatment	Outcomes
1	ETA	Numerous GNCB	Numerous *A. baumannii*	Carbapenem-resistant *A. baumannii*	*A. baumannii* (≥ 10^7^)	No detection	Colistin	Survived
2	ES	NOS	No growth	–	*P. aeruginosa* (10^6^)	CTX-M	Meropenem	Dead
3	ETA	Numerous GNR	Numerous *A.baumannii* Numerous *P. aeruginosa* Numerous *S. maltophilia*	XDR *A. baumannii* Non-MDR *P. aeruginosa* *S. maltophilia*	*A. baumannii* (≥ 10^7^), *P. aeruginosa* (10^5^)	No detection	Meropenem Levofloxacin	Dead
4	ETA	Moderate GNR	Numerous *P. aeruginosa*	Non-MDR *P. aeruginosa*	*P. aeruginosa* (10^6^) Human rhinovirus	No detection	Ciprofloxacin	Dead
5	ETA	Numerous GPC	No growth	–	No detection	No detection	Piperacillin- tazobactam	Survived
6	ES	Few GNR	Few *P. aeruginosa*	Non-MDR *P. aeruginosa*	*P. aeruginosa* (≥ 10^7^)	No detection	Piperacillin- tazobactam	Survived
7	ETA	Few GNR, few GPB	Few *K. pneumoniae* Few *S. maltophilia*	ESCR *K. pneumoniae* *S. maltophilia*	*K. pneumoniae* (10^6^)	CTX-M	Piperacillin- tazobactam, levofloxacin	Survived
8	ETA	Moderate GNR, few GPC	Few *P. aeruginosa*	Non-MDR *P. aeruginosa*	*P. aeruginosa* (10^6^)	No detection	Ciprofloxacin	Survived
9	ETA	Moderate GNR, moderate GNCB	Numerous *P. aeruginosa* Numerous *A. baumannii* Numerous *S. maltophilia*	Carbapenem-resistant *P. aeruginosa* XDR *A. baumannii* *S. maltophilia*	*P. aeruginosa* (≥ 10^7^) *A. baumannii* (10^6^) *K. pneumoniae* (10^4^)	OXA-48	Colistin, levofloxacin	Survived
10	ETA	Few GNR, few GNCB, few GPB	Numerous *A. baumannii* Numerous *K. pneumoniae*	XDR *A. baumannii* ESCR *K. pneumoniae*	*A. baumannii* (≥ 10^7^) *K. pneumoniae* (10^5^)	CTX-M	Colistin	Survived
11	ETA	Few GNR, few GPC	Few *A. baumannii*	Non-MDR *A. baumannii*	*A. baumannii* (10^4^)	No detection	Meropenem	Survived
12	ETA	Numerous GNR, few GPB	Numerous *K. pneumoniae* Few *A. baumannii* Few *P. aeruginosa*	ESCR *K. pneumoniae* Non-MDR *A. baumannii* Non-MDR *P. aeruginosa*	*K. pneumoniae* (10^6^) *A. baumannii* (10^5^) *P. aeruginosa* (10^4^)	CTX-M	Meropenem	Dead
13	ETA	Few GNCB	Moderate *A. baumannii*	XDR *A. baumannii*	*A. baumannii* (≥ 10^7^) Parainfluenza virus	No detection	Colistin	Survived
14	ETA	Few GNCB	Numerous *A. baumannii*	XDR *A. baumannii*	*A. baumannii* (≥ 10^7^) Influenza A	No detection	Colistin	Dead
15	ETA	Numerous GPC	Moderate *S. aureus*	Methicillin susceptible *S. aureus*	*E. coli* (10^5^)	No detection	Piperacillin- tazobactam	Dead
16	ETA	Few GPB	No growth	–	No detection	No detection	Piperacillin- tazobactam	Dead
17	ETA	Few GPB	Numerous *A. baumannii*	XDR *A. baumannii*	*A. baumannii* (10^6^)	No detection	Colistin	Dead
18	ES	Mixed organisms	Oral microbiota	–	*S. aureus* (10^6^)	No detection	Piperacillin- tazobactam	Dead
19	ETA	Moderate GNCB, few GPC, moderate GPB	Numerous *A. baumannii*	XDR *A. baumannii*	*A. baumannii* (≥ 10^7^) *P. mirabilis* (10^6^) *K. pneumoniae* (10^5^) *P. aeruginosa* (10^4^)	No detection	Meropenem	Survived
20	ETA	Few GPB	Few *P. aeruginosa* Few *E. coli* Few *S. aureus*	Non-MDR *P. aeruginosa* ESCR *E. coli* Methicillin resistant *S. aureus*	*P. aeruginosa* (10^6^) *E. coli* (10^4^) *S. aureus* (10^4^)	CTX-M, *MecA/C* and MREJ	Piperacillin- tazobactam, vancomycin	Survived
21	ETA	Numerous GNR	Numerous *P. aeruginosa* Numerous *S. maltophilia*	Carbapenem-resistant *P. aeruginosa* *S. maltophilia*	*P. aeruginosa* (≥ 10^7^) *A. baumannii* (10^5^) *K. pneumoniae* (10^5^)	CTX-M, OXA-48	Colistin, levofloxacin	Dead
22	ETA	Few GPB	Few *P. aeruginosa*	Carbapenem-resistant *P. aeruginosa*	*P. aeruginosa* (≥ 10^7^) *A. baumannii* (10^5^) *S. aureus* (10^4^)	*MecA/C* and MREJ	Ceftazidime	Survived
23	ETA	Moderate GNR, moderate GPB, few GPC	Moderate *A. baumannii*	Non-MDR *A. baumannii*	*A. baumannii* (≥ 10^7^)	No detection	Piperacillin- tazobactam	Dead
24	ETA	Numerous GNCB, numerous GPB	Numerous *A. baumannii* Numerous *K. pneumoniae*	XDR *A. baumannii* Non-MDR *K. pneumoniae*	*A. baumannii* (≥ 10^7^) *K. pneumoniae* (10^6^)	No detection	Colistin	Dead
25	ETA	Moderate GNCB, few GNR	Moderate *A. baumannii* Few *P. aeruginosa*	XDR *A. baumannii* Non-MDR *P. aeruginosa*	*A. baumannii* (≥ 10^7^) *P. aeruginosa* (≥ 10^7^)	No detection	Colistin	Dead
26	ETA	Few GNR	Numerous *P. aeruginosa*	Non-MDR *P. aeruginosa*	*P. aeruginosa* (≥ 10^7^) *A. baumannii* (≥ 10^7^)	CTX-M	Piperacillin- tazobactam	Survived
27	ETA	Numerous GNCB, numerous GNR, few GPB	Numerous *A. baumannii* Numerous *P. aeruginosa* Numerous *S. maltophilia*	Carbapenem-resistant *A. baumannii* Carbapenem-resistant *P. aeruginosa* *S. maltophilia*	*A. baumannii* (≥ 10^7^) *P. aeruginosa* (≥ 10^7^) *K. pneumoniae* (10^5^) *E. coli* (10^5^)	CTX-M	Colistin, levofloxacin	Dead
28	ETA	Moderate GPC	Moderate *S. aureus*	Methicillin susceptible *S. aureus*	*S. aureus* (10^6^)	No detection	Cefazolin	Survived
29	ETA	Numerous GNR	Numerous *P. aeruginosa* Numerous *S. maltophilia*	Carbapenem-resistant *P. aeruginosa* *S. maltophilia*	*P. aeruginosa* (≥ 10^7^)	No detection	Ceftazidime, levofloxacin	Survived
30	ETA	Numerous GNCB, few GNR	Numerous *A. baumannii* Numerous *S. maltophilia*	XDR *A. baumannii* *S. maltophilia*	*A. baumannii* (≥ 10^7^) *K. pneumoniae* (10^5^) *E. coli* (10^4^)	CTX-M, OXA-48	Colistin, levofloxacin	Dead
31	ES	NOS	Oral microbiota	–	*E. coli* (10^4^)	No detection	Piperacillin- tazobactam	Survived
32	ETA	Numerous GNCB	Numerous *A. baumannii* Numerous *S. maltophilia*	XDR *A. baumannii* *S. maltophilia*	*A. baumannii* (≥ 10^7^) *K. pneumoniae* (10^5^) *P. aeruginosa* (10^5^) *S. aureus* (10^4^)	CTX-M, OXA-48, *MecA/C* and MREJ	Piperacillin- tazobactam, vancomycin	Dead
33	ETA	Numerous GNCB	Numerous *A. baumannii* Few *P. aeruginosa*	XDR *A. baumannii* Non-MDR *P. aeruginosa*	*A. baumannii* (≥ 10^7^) *P. aeruginosa* (10^4^)	No detection	Colistin, levofloxacin	Dead
34	ETA	Numerous GNR	Numerous *K. pneumoniae*	Non-MDR *K. pneumoniae*	*K. pneumoniae* (≥ 10^7^) *A. baumannii* (≥ 10^7^) *E. coli* (≥ 10^7^) *S. aureus* (10^6^) *E. cloacae* complex (10^5^) *P. aeruginosa* (10^5^)	CTX-M, OXA-48, IMP	Ceftriaxone	Dead
35	ETA	Few GNR, few GPB	Moderate *P. aeruginosa*	Non-MDR *P. aeruginosa*	*P. aeruginosa* (≥ 10^7^) *A. baumannii* (≥ 10^7^) *K. pneumoniae* (10^5^) *S. aureus* (10^5^)	NDM, VIM	Piperacillin- tazobactam	Survived
36	ETA	NOS	Few *A. baumannii*	XDR *A. baumannii*	*A. baumannii* (10^6^)	No detection	Colistin	Dead
37	ETA	Moderate GPB, few GPC	Numerous *P. aeruginosa*	Non-MDR *P. aeruginosa*	*P. aeruginosa* (10^6^) *A. baumannii* (10^5^)	No detection	Levofloxacin	Survived
38	ETA	Moderate GNCB, few GNR	Numerous *A. baumannii*	XDR *A. baumannii*	*A. baumannii* (≥ 10^7^)	NDM	Colistin, meropenem	Dead
39	ETA	Numerous GNCB, few GPB	Numerous *A. baumannii*	XDR *A. baumannii*	*A. baumannii* (≥ 10^7^) *K. pneumoniae* (10^5^) *P. aeruginosa* (10^4^)	CTX-M, NDM, OXA-48	Colistin, meropenem	Dead
40	ETA	NOS	Few *S. maltophilia*	*S. maltophilia*	*S. agalactiae* (10^6^) *K. pneumoniae* (10^4^)	NDM, OXA-48	Levofloxacin	Survived

AMR, antimicrobial resistant; AST, antimicrobial susceptibility testing; BFPP, BioFire FilmArray pneumonia panel; ES, expectorated sputum; ESCR, extended-spectrum cephalosporin-resistant; ETA, endotracheal aspirate; GNCB, gram-negative coccobacilli; GNR, gram-negative rod; GPB, gram-positive bacilli; GPC, gram-positive cocci; NOS, no organism seen; MDR, multidrug-resistant; XDR, extensively drug-resistant.

### Baseline characteristics and results of conventional microbiological testing

Of the 40 subjects, 21 (52.5%) were male, and the mean age was 71 ± 16. Hypertension (65.0%), chronic kidney disease (50.0%), diabetes mellitus (45.0%), and cardiovascular disease (45.0%) were the most common comorbidities (**Table 2**). More than half of the subjects (57.5%) had been hospitalized in the preceding three months, and 17 (42.5%) had received antimicrobials. Of those who received antimicrobial therapy, the cephalosporin group (22.5%) and beta-lactam/beta-lactamase inhibitors (15.0%) were the most frequently administered. Most subjects (90.0%) were admitted to general medical wards. The median onset of HAP/VAP diagnosis was 10.5 (5, 21.5) days after admission; 35 (87.5%) required mechanical ventilatory support, and 21 (52.5%) died. Conventional cultures identified 54 organisms from 40 samples; 17 samples detected at least two different species, and three samples had no organism growth. Gram Negatives, such as *A. baumannii* (37.0%), *P. aeruginosa* (29.6%), *S. maltophilia* (16.7%), and *K. pneumoniae* (9.3%), were the most common etiologies. Among Gram-positive organisms, only *S. aureus* was identified (5.6%). MDR, XDR, and carbapenem-resistant (68.6%) and non-MDR (31.4%) phenotypes were the AST profiles among Gram-negative bacteria. However, the AST profiles varied widely based on the bacterial species. For example, among 20 A*. baumannii*, 15 had XDR, and two had carbapenem-resistant phenotypes. Among 16 P*. aeruginosa*, 11 had non-MDR, and five had carbapenem-resistant phenotypes. Among six *Enterobacterales*, ESCR phenotypes were found in three *K. pneumoniae* and one *Escherichia coli* isolates. Nine *S. maltophilia* isolates were susceptible to trimethoprim-sulfamethoxazole, and 89% were susceptible to levofloxacin (data not shown).

**Table 2:** Baseline characteristics and the conventional microbiological results of study subjects with hospital-acquired pneumonia (HAP) and ventilator-associated pneumonia (VAP).

Characteristics	Total (n = 40)
Male, n (%)	21 (52.5)
Mean age ± SD, years	71 ± 16
**Underlying disease**, n (%)	
Hypertension	26 (65.0)
Chronic kidney disease	20 (50.0)
Diabetes mellitus	18 (45.0)
Cardiovascular disease	18 (45.0)
Heart failure	7 (17.5)
Chronic lung disease	6 (15.0)
Malignancy	5 (12.5)
Liver disease	2 (5.0)
**Past medical history**, n (%)	
Previous antimicrobial administration in the last 3 months	17 (42.5)
**Type of previous antimicrobial administration**	
Penicillin or ampicillin group	3 (7.5)
Carbapenem group	2 (5.0)
Cephalosporin group	9 (22.5)
Beta-lactam/beta-lactamase inhibitors	6 (15.0)
Others (aminoglycoside, fluoroquinolone, and colistin)	3 (7.5)
Previous hospitalization in the last 3 months	23 (57.5)
Recent undergoing medical procedure	6 (15.0)
**Cause of hospitalization**, n (%)	
Infection-related	19 (47.5)
Non-infection related	21(52.5)
**Ward/unit of hospitalization**, n (%)	
ICU	3 (7.5)
General medical ward	36 (90.0)
General surgical ward	1 (2.5)
**Type of specimen sampling**, n (%)	
Endotracheal aspiration	36 (90.0)
Expectorated sputum	4 (10.0)
Median onset before HAP/VAP diagnosis (IQR), days	10.5 (5, 21.5)
Requiring mechanical ventilatory support, n (%)	35 (87.5)
**Conventional microbiological results**	
Single organism, n (%)	20 (50.0)
≥ 2 organisms, n (%)	17 (42.5)
**Type of etiologic agent**, n (%)	54
*Acinetobacter baumannii*	20 (37.0)
*Pseudomonas aeruginosa*	16 (29.6)
*Stenotrophomonas maltophilia*	9 (16.7)
*Klebsiella pneumoniae*	5 (9.3)
*Staphylococcus aureus*	3 (5.6)
*Escherichia coli*	1 (1.9)
**Antimicrobial susceptibility profiles of isolates**, n (%)	
Total	54
Non-MDR *K. pneumoniae*	2 (3.7)
Extended-spectrum cephalosporin-resistant *K. pneumoniae*	3 (5.6)
Extended-spectrum cephalosporin-resistant *E. coli*	1 (1.9)
Non-MDR *P. aeruginosa*	11 (20.4)
Carbapenem-resistant *P. aeruginosa*	5 (9.3)
Non-MDR *A. baumannii*	3 (5.6)
Carbapenem-resistant *A. baumannii*	2 (3.7)
XDR *A. baumannii*	15 (27.8)
Methicillin-susceptible *S. aureus*	2 (3.7)
Methicillin-resistant *S. aureus*	1 (1.9)
*S. maltophilia*	9 (16.7)
**Treatment outcomes**, n (%)	
Survived	19 (47.5)
Dead	21 (52.5)

ICU, intensive care unit; HAP, hospital-acquired pneumonia; IQR, interquartile range; MDR, multidrug-resistant; VAP, ventilator-associated pneumonia; XDR, extensively drug-resistant.

### Targeted organisms and AMR gene detection by the BFPP assay

The BFPP assay identified 81 organisms from 40 samples; 23 samples detected at least two different targets, and two samples had no targets detected (**Table 3**). The most common bacterial target detected was *A. baumannii* (32.5%), followed by *P. aeruginosa* (26.3%), *K. pneumoniae* (17.5%), and *S. aureus* (8.8%). Miscellaneous bacterial targets were also detected by the BFPP, such as *Proteus* spp., *Enterobacter* spp., and *Streptococcus agalactiae.* Overall, the assay was able to detect 28 AMR genes, which were the most prevalent among Gram-negative organisms, for example, CTX-M (42.9%), which is associated with ESCR, and OXA-48-like (25.0%) and NDM (14.3%), which is associated with carbapenem resistance. In addition, three *mecA/C* and MREJ genes that are associated with methicillin resistance were detected in corresponding *S. aureus* detections.

**Table 3:** Targeted organisms and antimicrobial resistance gene detection by the BioFire FilmArray pneumonia panel plus assay.

Target	Total
**Bacterial targets**, n (%)	40
No detection	2 (5.0)
Single organism	15 (37.5)
≥ 2 organisms	23 (57.5)
**Type of target detected**, n (%)	80
*A. baumannii*	26 (32.5)
*P. aeruginosa*	21 (26.3)
*K. pneumoniae*	14 (17.5)
*S. aureus*	7 (8.8)
*E. coli*	6 (7.5)
*Proteus* spp.	1 (1.3)
*Enterobacter* spp.	1 (1.3)
*S. agalactiae*	1 (1.3)
Viral targets^a^	3 (3.8)
**Antimicrobial-resistant gene targets**, n (%)	
**Total**	28
CTX-M	12 (42.9)
OXA-48-like	7 (25.0)
NDM	4 (14.3)
VIM	1 (3.6)
IMP	1 (3.6)
*mecA/C* and MREJ	3 (10.7)

a Co-detection of viral targets— Rhinovirus (1), Parainfluenza (1), and Influenza A viruses (1).

### Diagnostic efficacy and level of agreement of the BFPP assay for diagnosing etiology of HAP/VAP

The four most common HAP/VAP etiologies were analyzed. Among Gram-negative species, the BFPP assay exhibited high diagnostic sensitivities with good agreements for detecting *A. baumannii* [sensitivity of 100% (83-100% CI), *κ* = 0.70 (0.49-0.91 CI)] and *P. aeruginosa* [sensitivity of 100% (79-100% CI), *κ* = 0.75 (0.56-0.95 CI)], whereas the assay had high diagnostic sensitivities with moderate agreements for detecting *K. pneumoniae* [sensitivity of 100% (48-100% CI), *κ* = 0.42 (0.15-0.69)]. For *S. aureus* detection, the BFPP assay showed moderate diagnostic sensitivities [67% (9-99% CI)] with fair agreements [*κ* = 0.33 (-0.65-0.73 CI)] (**Table 4**).

**Table 4:** Sensitivity, specificity, positive predictive value, negative predictive value, and Cohen’s kappa coefficient (*κ*) of BioFire FilmArray pneumonia panel plus assay to detect common individual etiologies of hospital-acquired pneumonia and ventilator-associated pneumonia.

BFPP bacterial target (n)	Standard conventional culture		Sensitivity (%)(95% CI)	Specificity (%)(95% CI)	PPV (%)(95% CI)	NPV (%)(95% CI)	*κ*
	Positive (n)	Negative (n)					
***A. baumannii*** (26)							
Detected	20	6	100 (83-100)	70 (46-88)	77 (56-91)	100 (77-100)	0.70 (0.49-0.91)
Not detected	0	14					
***P. aeruginosa*** (21)							
Detected	16	5	100 (79-100)	79 (58-93)	76 (53-92)	100 (82-100)	0.75 (0.56-0.95)
Not detected	0	19					
***K. pneumoniae*** (14)							
Detected	5	9	100 (48-100)	74 (57-88)	36 (13-65)	100 (87-100)	0.42 (0.15-0.69)
Not detected	0	26					
***S. aureus*** (7)							
Detected	2	5	67 (9-99)	86 (71-96)	29 (4-71)	97 (84-99)	0.33 (-0.65-0.73)
Not detected	1	32					

BFPP, BioFire FilmArray pneumonia panel; CI, confidence interval; NPV, negative predictive value; PPV, positive predictive value; κ, Cohen’s kappa coefficient.

Seventy-four bacterial targets detected by the BFPP were compared to all bacteria (45) identified by the conventional cultures to evaluate the overall diagnostic efficacy of the BFPP assay (**Table 5**). We removed *S. maltophilia* (9) from the analysis because this species is not in the BFPP target panel. The BFPP had an overall sensitivity of 98% (88-100% CI), a specificity of 81% (74-87% CI), a positive predictive value of 60% (47-71% CI), and a negative predictive value of 99% (96-100% CI), with a *κ* of 0.64 (0.53-0.75 CI).

**Table 5:** Overall sensitivity, specificity, positive predictive value, negative predictive value, and Cohen’s Kappa coefficient (κ) of BioFire FilmArray Pneumonia Panel Plus assay to detect all identified etiologies of hospital-acquired pneumonia and ventilator-associated pneumonia.

BFPP bacterial target (n)	Standard conventional culture^a^		Sensitivity (%)(95% CI)	Specificity (%)(95% CI)	PPV (%)(95% CI)	NPV (%)(95% CI)	*κ*
	Positive (n)	Negative (n)				
**Overall** (74)^b^							
Detected	44	30	98 (88-100)	81 (74-87)	60 (47-71)	99 (96-100)	0.64 (0.53-0.75)
Not detected	1	125					

BFPP, BioFire FilmArray pneumonia panel; CI, confidence interval; NPV, negative predictive value; PPV, positive predictive value; κ, Cohen’s kappa coefficient.

a Only 45 bacteria species identified by the conventional cultures were included: S. maltophilia (9) was not included.

b Bacterial targets detected by the BFPP—*A. baumannii* (26), *P. aeruginosa* (21), *K. pneumoniae* (14), *S. aureus* (7), and *E. coli* (6) were included.

In addition, overall Gram stain findings were compared with standard conventional culture to evaluate diagnostic efficacy and agreement (**Supplementary Table 1**). Gram stain from respiratory samples exhibited moderate sensitivities (63.5%) with very low specificities (5.9%), and it had a substantially poor agreement (*κ* = -0.27) with the standard culture. We also analyzed the agreement of the BFPP assay compared with Gram stain findings as a non-reference standard (**Supplementary Table 2**). We removed gram-positive bacilli detected by Gram stain from the analysis since they are not in the BFPP target. Our study demonstrated that the BFPP had moderate positive percent agreement (54.5%). In contrast, it had no negative percent agreement (0%) and substantially poor concordance (*κ* = -0.18) when using respiratory sample Gram staining as the reference.

### Concordant and discordant results between the BFPP AMR gene detection and the phenotypic antimicrobial susceptibility testing profile

Of six *Enterobacterales*, five had an AST phenotype concordant with the BFPP AMR gene detection, and only one strain had a discordant phenotype because of the co-existence of other Gram-negative and other AMR gene targets detected (κ = 0.57). Of the 16 P*. aeruginosa*, seven strains had non-MDR phenotypic AST profiles concordant with no AMR gene detections, and nine had AST profiles discordant with the BFPP assay (κ < 0.00). Of 20 A*. baumannii*, two of three non-MDR phenotypes had concordant results with the BFPP tests. Among 17 A*. baumannii* with carbapenem-resistant and XDR phenotypes, 15 had phenotypes discordant with the BFPP tests, and only two strains with XDR phenotypes had concordant NDM gene detections (κ < 0.00). Only three *S. aureus* were detected; two strains had phenotypes concordant with the BFPP tests (κ not calculated) (**Table 6**).

**Table 6:** Number of concordant and discordant results of the AMR gene detection by BioFire FilmArray pneumonia panel plus assay compared to the phenotypic antimicrobial susceptibility testing profile of the bacterial isolate.

Phenotypic AST profile of the isolate (n)	Result of the BioFire Film Array Pneumonia Panel Plus assay		
	Concordant (n)	Discordant (n)	Detail of discordant results and dissociated AMR gene detection (n)
Non-MDR *K. pneumoniae* (2)	1^a^	1	Co-existence of other GN targets with CTX-M, IMP, and OXA-48 genes detected (1)
ESCR *K. pneumoniae* (3)	3^b^		
ESCR *E. coli* (1)	1^b^		
Non-MDR *P. aeruginosa* (11)	7^a^	4	Co-existence of other GN targets with CTX-M genes detected (3); co-existence of other GN targets with NDM and VIM genes detected (1)
Carbapenem-resistant *P. aeruginosa* (5)		5	No carbapenemase genes detected (2); co-existence of other GN targets with CTX-M genes detected (1); co-existence of other GN targets with CTX-M and OXA-48 genes detected (1); co-existence of other GN targets with OXA-48 genes detected (1)
Non-MDR *A. baumannii* (3)	2^a^	1	Co-existence of other GN targets with CTX-M genes detected (1)
Carbapenem-resistant *A. baumannii* (2)		2	No carbapenemase genes detected (1); co-existence of other GN targets with CTX-M genes detected (1)
XDR *A. baumannii* (15)	2^c^	13	No carbapenemase genes detected (9); co-existence of other GN targets with CTX-M genes detected (1); co-existence of other GN targets with CTX-M and OXA-48 genes detected (2); co-existence of other GN targets with OXA-48 genes detected (1)
MS *S. aureus* (2)	1^a^	1	*E. coli* was detected instead (1)
MR *S. aureus* (1)	1^d^		

AMR, antimicrobial resistant; AST, antimicrobial susceptibility testing; ESCR, Extended-spectrum cephalosporin-resistant: GN, Gram Negative; MDR, multidrug-resistant; MR, methicillin-resistant MS, methicillin-susceptible; XDR, extensively drug-resistant.

a No associated AMR gene target was detected.

b Only CTX-M gene was detected.

c Only NDM gene was detected (1); co-existence of other GN targets with CTX-M, NDM, and OXA-48 genes was detected (1).

d mecA/MecC and MREJ and co-existence of GN targets with CTX-M genes were detected.

## Discussion

Compared with conventional microbiological tests, the BFPP assay has an excellent performance in diagnosing the etiology of HAP/VAP. However, some bacterial species not included in the assay targets can limit the test sensitivity. We observed several discordant results with AMR gene detection that reduced the performance of the BFPP. Our subjects’ clinical characteristics differed from other studies (Lee et al., 2019; Webber et al., 2020; Mitton et al., 2021). Most were elderly (71 ± 16 years) with multiple comorbidities, and almost half had recent antimicrobial administrations and hospitalizations. HAP/VAP occurred late in the hospital stay (median 10.5 days), and almost 90% of subjects had a severe respiratory impairment that required mechanical ventilatory support. As a result, almost half of the subjects died. In other studies, subjects tended to be younger (45-65 years), had an onset of HAP/VAP earlier in the hospital stay, and were more likely to survive (Lee et al., 2019; Webber et al., 2020; Mitton et al., 2021; Kamel et al., 2022). Consistent with other studies, Gram-negative bacteria were the most common etiologic agents, but the species of gram-negative bacteria we detected were vastly different. Based on conventional cultures, *A. baumannii*, *P. aeruginosa*, *and S. maltophilia* were the three most common HAP/VAP etiologies. In contrast, other studies reported that *Enterobacterales*, particularly *K. pneumoniae*, and *P. aeruginosa* were more prevalent than *A. baumannii* (Lee et al., 2019; Webber et al., 2020; Kamel et al., 2022; Enne et al., 2022). In contrast with other reports, we observed that *S. aureus* was not a common cause of HAP/VAP (Webber et al., 2020; Yoo et al., 2020; Enne et al., 2022). *S. maltophilia* is an emerging cause of HAP/VAP in our institute. This agent usually causes HAP/VAP in individuals who have prolonged MV support or received prior broad-spectrum beta-lactam or carbapenem therapy (Insuwanno et al., 2020). However, the BFPP assay does not include this bacterial target which limits the diagnostic utility of the assay in patients with such risk factors. Thus, using the BFPP assay for diagnosing late HAP/VAP should consider the local prevalence of causative organisms in the individual institute. Multiple bacterial co-infections are frequently seen in mechanically ventilated individuals. Similar to a study by Lee et al., we observed that nearly 43% of subjects had multiple organisms in the same respiratory sample (Lee et al., 2019). The growth of multiple organisms makes it challenging to distinguish between colonization or actual infections clearly.

Compared with conventional cultures, the BFPP exhibited an overall sensitivity of 98% and a specificity of 81% to diagnose HAP/VAP etiology, a finding consistent with other studies (Yoo et al., 2020; Mitton et al., 2021; Kamel et al., 2022). In addition, the sensitivities of this assay did not change when common bacterial etiologies, such as *A. baumannii*, *P. aeruginosa*, and *K. pneumoniae*, were separately analyzed (**Table 4**). However, the positive results may not have been reliable since many bacterial targets detected by the assays could not be grown in conventional cultures. *K. pneumoniae* and *S. aureus* also exhibited a fair agreement by the *κ* coefficient when compared with conventional cultures. The BFPP assay has been reported to identify multiple bacterial targets in approximately 40-45% of samples (Lee et al., 2019; Webber et al., 2020; Kamel et al., 2022), but we found that almost 60% of our samples had multiple bacterial targets. It is not uncommon for the BFPP assay to detect several bacterial species that cannot be grown in conventional cultures (Buchan et al., 2020; Murphy et al., 2020; Webber et al., 2020; Mitton et al., 2021; Kamel et al., 2022). The substantial variability in the numbers of causative bacteria may explain this finding; for example, the smaller quantities may be missed on the culture plate due to an overgrowth of other bacteria, or the targets detected by the BFPP assay may not be viable organisms. Thus, bacterial targets detected by the BFPP assay should be carefully interpreted; clinical and conventional microbiological findings are still required before making a HAP or VAP diagnosis.

A good-quality respiratory sample Gram stain with numerous bacteria detected can support the diagnosis of HAP/VAP (Kalil et al., 2016). According to our study, Gram stain from a respiratory sample showed moderate diagnostic sensitivity to detect HAP/VAP etiology. However, it demonstrated inferior agreement with a standard culture similar to a previous study (O’Horo et al., 2012). Gram stain morphology may reflect respiratory pathogens and non-pathogenic bacteria colonized within the respiratory tract. Only potential pathogens isolated on cultures are selectively reported by our microbiology laboratory protocol, which could result in disagreement between those two tests. In addition, targets detected by the BFPP assay exhibited substantial discordance with organisms detected by Gram stain. More than half of patients received previous antimicrobial therapy before the HAP/VAP onset; prior antimicrobial therapy before sample collection may reduce the sensitivity of Gram stain to detect etiologic agents. Meanwhile, the BFPP assay had superior sensitivity. Thus, respiratory sample Gram stain is only adjunctive diagnostic testing. A good-quality respiratory sample Gram stain should be combined with clinical findings to diagnose HAP/VAP. Previous studies used several markers, such as the level of white blood cells reported on Gram stain, clinical pulmonary infection score (CPIS), procalcitonin level, and the level of bacterial DNA (copies/mL), to determine the clinical significance of the situation where the BFPP is positive while the conventional tests are negative (Kyriazopoulou et al., 2021; Rand et al., 2021a; Rand et al., 2021b). Only three (3.8%) viral targets were detected in our study; the role of viral pathogens in HAP or VAP is still unknown (Buchan et al., 2020).

There were only three *S. aureus* isolates; thus, the concordance between the BFPP and phenotypic AST could not be determined. However, the BFPP has shown good sensitivity (80-100%) and specificity (70-100%) to detect the *mecA/C* and MREJ from respiratory samples (Buchan et al., 2020; Murphy et al., 2020; Webber et al., 2020; Mitton et al., 2021). Of Gram-negative AMR genes detected by the BFPP assay, CTX-M (12) was the most common, followed by carbapenemase genes—OXA-48-like (7), NDM (4), VIM (1), and IMP (1). In our study, some gram-negative bacteria with AMR detections had discordant results. There are multiple potential causes of discordant results; co-existence of other bacterial targets and/or non-corresponding AMR genes detected or a finding of an antimicrobial-resistant profile by the phenotypic AST in the absence of AMR gene detection. CTX-M detection was widely distributed among *Enterobacterales* and non-*Enterobacterales* gram-negative organisms. We observed that the CTX-M detected by the assay had good concordance among *Enterobacterales* but was poorly concordant among *A. baumannii* and *P. aeruginosa* (**Table 6**). The CTX-M is generally found in *Enterobacterales* and other non-enteric gram-negative bacteria (Cantón et al., 2012). Thus, detection of CTX-M by the BFPP is not highly specific for an individual bacterial target, especially when multiple bacteria are simultaneously detected. Carbapenemase genes are predominantly found in *Enterobacterales*, whereas *A. baumannii* or *P. aeruginosa* resistant to carbapenem or several classes of antimicrobials may have other AMR mechanisms, such as non-OXA-48 typed carbapenemases, porin mutations, and efflux pump overexpression (Nordmann and Poirel, 2019). The other mechanisms are not currently included in the panel, which resulted in discordance between the phenotypic and genotypic testing. Thus, if the BFPP assay does not detect an AMR gene target, particularly in non-*Enterobacterales*, it does not always indicate the absence of phenotypic resistance. To determine the optimal antimicrobial therapy, we suggest that AMR gene detection results be carefully interpreted in combination with local epidemiological data and hospital antibiogram.

An increased cost should be in consideration before clinical utilization of the BFPP assay for routine microbiological testing since conventional culture and antimicrobial susceptibility are still required to confirm the phenotype. Previous studies reported multiplex PCR assays, particularly for respiratory viruses, are more cost-effective than traditional microbiological diagnostic testing (Mahony et al., 2009; Dundas et al., 2011). Other studies demonstrated that approximately 60-70% of HAP patients receive timely antimicrobial modification and optimization when the real-time BFPP results are available (Buchan et al., 2020; Erich et al., 2022). In addition, 60.7% of patients received antimicrobial de-escalation based on the results of the BFPP assay (Erich et al., 2022). However, studies of cost-effectiveness, including the clinical impact on patient management and outcomes, antimicrobial stewardship, and infection prevention of the BFPP assay, have been scarcely evaluated and have still been unmet diagnostic needs (Hanson et al., 2020).

Our study has some limitations. Our study sample size was small, and some bacterial species were not detected in sufficient numbers to assess the efficacy of the BFPP. There were substantial variations and disagreements between the phenotypic AST and genotypic testing such that the performance of the AMR gene detection could not be completely determined. We did not resolve discrepancies between the phenotypic tests and the BFPP assay by further conventional testing, and using only clinical evaluation might introduce bias. In addition, the study did not evaluate the BFPP on clinical outcomes and patient management. A more extensive prospective study, including clinical evaluation and costs, is warranted to confirm the clinical utility of this assay.

## Conclusions

In the setting where MDR or XDR organisms are an emerging cause of HAP or VAP, the BioFire FilmArray pneumonia panel has a high diagnostic sensitivity to detect a causative organism of HAP/VAP. However, *S. maltophilia*, which is increasingly prevalent, is not a targeted organism and can be missed by the panel. In addition, conventional testing and antimicrobial-resistant gene detection may be discordant when multiple bacteria are detected or the causative bacteria carry other AMR mechanisms. Particularly with non-*Enterobacterales* gram-negative bacteria, AMR gene detection results should be carefully interpreted in combination with local epidemiological data and hospital antibiogram. Understanding the limitations of the BFPP test is essential to optimize its clinical use.

## Data availability statement

The raw data supporting the conclusions of this article will be made available by the authors, without undue reservation.

## Ethics statement

The studies involving human participants were reviewed and approved by The Scientific Ethics Committee, Siriraj Institutional Review Board (COA Si 673/2020). The patients/participants provided their written informed consent to participate in this study.

## Author contributions

AJ, the corresponding author, was responsible for the study’s conceptualization and design, the data acquisition, the drafting of the manuscript, and the final revision. SH and NH participated in the conceptualization and design of the study, methodology, and manuscript drafting. SP participated in data acquisition, data analysis, and manuscript drafting. SP participated in data analysis and manuscript drafting. All authors contributed to the article and approved the submitted version.

## Funding

This study was funded by Siriraj Grants in Research and Development (grant number R016431007). Dr. Anupop Jitmuang also receives a Chalermphrakiat Grant from the Faculty of Medicine Siriraj Hospital, Mahidol University.

## Acknowledgments

We express our sincere thanks to Ms. Archiraya Pattama, Ms. Juthamas Buahom, Ms. Sirikarn Athipanyasil, and the staff at the Molecular Diagnostic Unit, Department of Microbiology, Siriraj Hospital, for their assistance with the microbiological data in this study, and Ms. Khemajira Karaketklang for helping with the data analyses.

## Conflict of interest

The authors declare that the research was conducted in the absence of any commercial or financial relationships that could be construed as a potential conflict of interest.

## Publisher’s note

All claims expressed in this article are solely those of the authors and do not necessarily represent those of their affiliated organizations, or those of the publisher, the editors and the reviewers. Any product that may be evaluated in this article, or claim that may be made by its manufacturer, is not guaranteed or endorsed by the publisher.
