# Supplementary material for: A multiplex pneumonia panel for diagnosis of hospital-acquired and ventilator-associated pneumonia in the era of emerging antimicrobial resistance
Source: Front Cell Infect Microbiol. 2022 Oct 12;12:977320. doi: 10.3389/fcimb.2022.977320 (PMC9597303; doi:10.3389/fcimb.2022.977320)
Supplement: Supplementary file 1 [file Table_1.docx]

**Supplementary Table**

**Supplementary Table 1**. Overall sensitivity, specificity, positive predictive value, negative predictive value, and Cohen's Kappa coefficient (κ) of Gram stain findings compared with standard conventional culture from respiratory samples

| **Gram stain finding** (n) | **Standard conventional**  **culture** | | **Sensitivity** (%)  (95% CI) | **Specificity** (%)  (95% CI) | **PPV** (%)  (95% CI) | **NPV** (%)  (95% CI) | *κ* |
| --- | --- | --- | --- | --- | --- | --- | --- |
|  | **Positive**  (n) | **Negative**  (n) |  |  |  |  |  |
| **Overall** (80) |  |  |  |  |  |  |  |
| Detected | 40^a^ | 23^b^ | 63.5  (50.4-100) | 5.9  (0.1-28.7) | 71.4  (57.8-82.7) | 4.2  (0.1-21.1) | -0.27  (-0.43 to  -0.11) |
| Not detected | 16^c^ | 1^d^ |  |  |  |  |  |

Abbreviations: CI, confidence interval; GNCB, gram-negative coccobacilli; GNR, gram-negative rod; GPB, gram-positive bacilli; GPC, gram-positive cocci; NPV, negative predictive value; PPV, positive predictive value; κ, Cohen’s kappa coefficient

^a^ Gram stain findings were seemingly concordant with standard culture results; for example, GNCB morphology detected by Gram stain were likely *A*. *baumannii* identified by cultures; GNR morphology detected by Gram stain were likely *Enterobacterales*, *P. aeruginosa*, or *S. maltophilia* identified by cultures; GPC detected by Gram stain were likely *S*. *aureus* identified by cultures; and GPB detected by Gram stain were likely potentially pathogenic gram-positive bacilli identified by cultures.

^b^ Gram stain findings detected GNCB, GNR, GPC, or GPB, but standard culture results were negative for those organisms detected by Gram stain.

^c^ Gram stain findings revealed no GNCB, GNR, GPC, or GPB, but standard culture results were positive for those organisms not detected by Gram stain.

^d^ Gram stain findings revealed no organism seen in concordance with standard culture results negative.

**Supplementary Table 2**. Agreement and Cohen’s Kappa coefficient (κ) of BioFire FilmArray Pneumonia Panel Plus assay compared with Gram stain finding from respiratory samples

| **BFPP bacterial target** (n) | **Gram stain finding**^a^ | | **PPA** (%)  (95% CI) | **NPA** (%)  (95% CI) | *κ* |
| --- | --- | --- | --- | --- | --- |
|  | **Detected** (n) | **Not detected** (n) |  |  |  |
| **Overall** (87) |  |  |  |  |  |
| Detected | 42^b^ | 35^c^ | 54.5  (42.8-65.9) | 0  (0-30.8) | -0.18  (-0.32 to  -0.05) |
| Not detected | 10^d^ | 0^e^ |  |  |  |

Abbreviations: BFPP, BioFire FilmArray pneumonia panel; CI, confidence interval; GNCB, gram-negative coccobacilli; GNR, gram-negative rod; GPC, gram-positive cocci; NPA, negative percent agreement; PPA, positive percent agreement; κ, Cohen’s kappa coefficient

^a^ Gram-positive bacilli detected by Gram stain were not included in the analysis since they are not in the BFPP target.

^b^ Bacterial targets detected by the BFPP seemingly agreed with Gram stain findings; for example, *A*. *baumannii* detected by BFPP were likely GNCB morphology detected by Gram stain; *Enterobacterales* or *P*. *aeruginosa* detected by BFPP were likely GNR morphology detected by Gram stain; *S*. *aureus* or *Streptococcus* spp. detected by BFPP were likely GPC morphology detected by Gram stain.

^c^ The BFPP detected bacterial targets, but Gram stain findings revealed no organism morphologically compatible with individual BFPP targets.

^d^ The BFPP did not detect bacterial targets, but Gram stain findings detected GNCB, GNR, or GPC morphology.

^e^ The BFPP did not detect bacterial targets in agreement with no organism identified by Gram staining.
